# A Modified Beagle Model of Inducible Atrial Fibrillation Using a Right Atrium Pacemaker

**DOI:** 10.21470/1678-9741-2019-0363

**Published:** 2020

**Authors:** Song Yang, Bo Mei, Hai Liu, Wei Li, Chao-qun Wang, Mei Yang, Yuan Yue, Zhong-kai Wu

**Affiliations:** 1 Cardiothoracic Surgery Intensive Care Unit of First Affiliated Hospital of Sun Yat-sen University, Guangzhou, People’s Republic of China.; 2 Key Laboratory on Assisted Circulation, Ministry of Health, Guangzhou, People’s Republic of China.; 3 Department of Cardiovascular Surgery, Affiliated Hospital of North Sicuan Medical College, Nanchong, Sicuan, People’s Republic of China.; 4 Cardiac Surgery Department of First Affiliated Hospital of Zhengzhou University, Zhengzhou, People’s Republic of China.; 5 Ultrasonics Department of First Affiliated Hospital of Sun Yat-sen University, Guangzhou, People’s Republic of China.; 6 Second Cardiac Surgery Department of First Affiliated Hospital of Sun Yat-sen University, Guangzhou, People’s Republic of China.; 7 Yuexiu Meihua Community Health Service Center, Guangzhou, People’s Republic of China.

**Keywords:** Atrial Fibrillation, Animal Model, Mitral Valve Insufficiency, Immunohistochemistry, Real-Time Polymerase Chain Reaction, Heart Atria, Echocardiography

## Abstract

**Objective:**

To modify the chronic atrial fibrillation of atrial tachycardia pacing in beagles with a homemade pacemaker placed outside the body and to evaluate connective tissue growth factor and fibrosis of atrial tissue in our modified atrial tachycardia pacing beagle model.

**Methods:**

Twelve adult beagles of either sex were randomly divided into an atrial tachycardia pacing group and a control group (n=6 in each group). We performed the temporary pacemaker implantation at the right atrial appendage and put the pacemaker into the pocket of dog clothing in the atrial tachycardia pacing group. After eight weeks of atrial tachycardia pacing, the electrocardiography, transthoracic echocardiography, hematoxylin-eosin staining, and Masson’s staining of the right atrial appendages were performed along with the immunohistochemistry, quantitative real-time polymerase chain reaction, and Western blot analysis of connective tissue growth factor, collagen I, and collagen III.

**Results:**

In the atrial tachycardia pacing group, atrial fibrillation was induced in five beagles (83.3%); the left atrium enlarged significantly; more canines had mitral regurgitation; and the Masson’s staining, quantitative real-time polymerase chain reaction, and Western blot results demonstrated more obvious fibrosis of the left atrium.

**Conclusion:**

The modified beagle model of atrial fibrillation using a right atrium pacemaker outside the body was effective, increased connective tissue growth factor and collagen I messenger ribonucleic acid overexpression, and induced atrial fibrosis.

**Table:** 

Abbreviations, acronyms & symbols				
AF	= Atrial fibrillation		LAESA	= Left atrial end-systolic area
ANOVA	= Analysis of variance		LSD	= Least significant difference
ATP	= Atrial tachycardia pacing		LVED	= Left ventricular end-diastolic diameter
CCN2	= Cellular communication network 2		LVEF	= Left ventricular ejection fraction
CHF	= Congestive heart failure		mRNA	= Messenger ribonucleic acid
CTGF	= Connective tissue growth factor		PCR	= Polymerase chain reaction
CVO	= Chronic volume overload		qRT-PCR	= Quantitative real-time polymerase chain reaction
ECG	= Electrocardiography		RA	= Right atrium
ERP	= Effective refraction period		RHD	= Rheumatic heart disease
GAPDH	= Glyceraldehyde-3-phosphate dehydrogenase		RNA	= Ribonucleic acid
HE	= Hematoxylin-eosin		SD	= Standard deviation
IHC	= Immunohistochemistry		SR	= Sinus rhythm
LA	= Left atrium		TGFβ	= Transforming growth factor beta
LAEDA	= Left atrial end-diastolic area		TTE	= Transthoracic echocardiography

## INTRODUCTION

Atrial fibrillation (AF) is one of the most common sustained cardiac arrhythmias with a relevant effect on mortality and morbidity, especially in the elderly^[1]^. Subclinical episodes of AF occur frequently in type 2 diabetic patients and have been associated with a significantly increased risk of stroke^[2]^. Until now, many studies showed that the electrical remodelling and structural remodelling were the chief pathophysiological mechanisms. Additionally, autonomic dysfunction was thought to be associated with brief episodes of AF^[3]^. The atrial structural abnormalities included fibrosis^[4]^, dilation, ischaemia, infiltration, and hypertrophy^[5]^. The animal models of AF can be divided into acute AF models and chronic AF models^[6]^. The chronic AF models include the atrial tachycardia pacing (ATP) model, congestive heart failure (CHF) model, sterile pericarditis model, and chronic volume overload (CVO) model^[7]^. Because most patients have combined permanent AF, we chose the ATP AF canine model to perform the research. We have made some modifications to the common ATP AF model in order to understand the effect of this modified model.

AF occurs in a higher percentage as an idiopathic arrhythmic atrial disease, which may be associated with atrial fibrosis and enlargement that may be due to epigenetic modifications involving different molecular adaptive pathways^[8]^. This remodeling may also involve different and numerous inflammatory molecules that are related to AF pathogenesis, which worsen after a therapeutic approach^[9]^. In our department, more than 50% of patients had rheumatic heart disease (RHD), of which over 50% of those patients have permanent AF. We observed that the atrium was thickened and there was fibrosis from the pathological results; as we knew, fibrosis could be one reason for AF. Some researchers^[10]^ used delayed enhancement magnetic resonance imaging to assess the degree of atrial disease based on fibrotic changes and confirmed that every patient with AF possesses some degree of atrial fibrotic changes that vary between minimal and severe or extensive, including patients with stand-alone AF. Connective tissue growth factor (CTGF), also known as cellular communication network 2 (CCN2), is a member of the CCN family as a strong profibrotic factor^[11]^. CTGF is widely expressed during development and modulation of cell adhesion, proliferation, survival, migration, and extracellular matrix production in diverse cell types^[12]^. The CTGF overexpression was a hallmark of fibrosis in multiple tissues, including the skin, liver, heart^[13]^, lung, and kidney, and it was widely thought to be required to mediate the profibrotic effects of transforming growth factor beta (TGFβ)^[14]^. CTGF might be a new target for treatment of fibrosis. We wanted to know about CTGF and fibrosis of atrial tissue in our modified ATP beagle model.

## METHODS

### Experimental Groups

A total of 12 beagles (Guangdong Qianyan Biological Science and Technology Co., LTD) of either sex, weighing 12-17 kg, were allocated into two groups, including the control and ATP groups. The random method was the envelope method. All animal care and experiments were performed in accordance with the guide for the care and use of animals of the Laboratory Animal Center of Sun Yat-sen University. Experiments were performed with the permission of the local committee on animal experiments.

### Modified Animal Model

Beagles were anaesthetized with sodium pentobarbital (30 mg/kg) through the auricular veins and intubated. Then, we obtained blood samples and performed electrocardiography (ECG) (traced by Nihon Kohden ECG-9020P and an electrophysiological recorder MP150, Biopac Systems, California, USA) and transthoracic echocardiography (TTE) (General Electric Company, GE Vivid *i*). After the right lateral thoracotomy, an electrode (Flexon Multifilament Temporary Cardiac Pacing Lead, Covidien, China) was sewn onto the epicardial surface of the right atrial appendage for stimulation. Another electrode was sewn onto the subcutaneous tissue. The pericardium and thorax were closed. Two electrode lines were penetrated out the skin on the back and connected with the pacemaker (an output of 6 V with a 1.0 ms pulse duration, 400 bpm, Guangzhou Academy of Sciences, China). Cefuroxime was used during the operations. The pacemakers were put into waterproof bags and fixed in the pocket of clothes for the dogs. The beagles were sent back to the animal centre and the stitches were removed seven days later under anesthesia. At the same time, we turned on the pacemakers and set them at 400 bpm. After eight weeks of the ATP, ECG, TTE, and blood samples were obtained. Atrial tissue samples were harvested from the right atrial free wall and immersed in 10% neutral-buffered formalin solution for histological analysis, and other samples were snap-frozen in liquid nitrogen at -80 °C for Western blot analysis. The control groups were the same, but without pacemakers. We used sodium pentobarbital for anaesthesia when doing some invasive operations or detection, such as placement of the pacemaker or the electrophysiological examination. AF was induced by S1S2 (250 ms, 300ms, and 350 ms) and burst (600 bpm). The end points were eight weeks after the ATP, and all the beagles were sacrificed.

### Histology

Tissue samples were sliced at 5-µm thickness and stained with hematoxylin-eosin (HE) and Masson’s stainings. The microscopic images were scanned into a personal computer and quantitatively analysed with Caseviewer (3DHISTECH, Budapest, Hungary). Interstitial fibrosis was quantified on the basis of a colour discrimination algorithm and expressed as a percentage of the reference tissue area. The mean value of the three fields in each section was used for the analysis. Histological examination was performed by the investigators who were blinded to treatment assignment.

### Real-Time Polymerase Chain Reaction Analysis

Ribonucleic acid (RNA) was isolated from the atrial tissue using the TRIzol reagent (Wuhan Servicebio Technology Co., China), as directed by the manufacturer (Omega Bio-Tek Inc., Norcross, Georgia, USA, E.Z.N.A.® Total RNA kit). The messenger ribonucleic acid (mRNA) concentrations in tissue samples were evaluated using the real-time polymerase chain reaction (PCR) assay. All reactions were performed in triplicate, and glyceraldehyde-3-phosphate dehydrogenase (GAPDH) served as an internal control. The results were quantified as Ct values, where Ct is defined as the threshold cycle of PCR at which the amplified product is first detected and expressed as the ratio of target to control. The expression levels of CTGF mRNA, collagen I mRNA, and collagen III mRNA were quantified using real-time PCR. The primer sequences for the target genes are as follows (5'-3'): induced CTGF mRNA Fw: GCATCTTCGGTGGTACGGTGTA, Rev: TGGACCAGGCAGTTGGCTCTA; collagen I mRNA Fw: CCAAAGAAGCCTTGCCATC, Rev: CACGCGTTCCCCAAATCC; collagen III mRNA Fw: CTGGAGGATGGTTGCACG, Rev: GGACCACCAATGTCATAGG; and GAPDH Fw: CTCGCTTCGGCAGCACA, Rev: AACGCTTCACGAATTTGCGT.

### Western Blot Analysis

The expression levels of collagen I, collagen III, and CTGF proteins were assayed by Western blot. Immunoblotting was performed as previously described. In brief, solubilized protein was separated using electrophoresis and transferred to nitrocellulose membranes. Nonspecific binding was blocked by incubation in 5% milk in tris-buffered saline with Tween 20. The membranes were probed with specific antibodies and subsequently incubated with horseradish peroxidase-conjugated secondary antibodies.

### Statistical Analysis

All quantitative data were expressed as mean ± standard deviation (SD). Statistical comparisons among groups were performed by one-way analysis of variance (ANOVA). If significant effects were indicated by ANOVA, a least significant difference (LSD) *t*-test was used to evaluate the significance of differences between individual mean values. A two-tailed *P*<0.05 was considered significant (IBM SPSS Statistics software, version 23, Chicago, Illinois, USA).

## RESULTS

### Atrial Fibrillation Duration and Inducibility

**Fig. 1:**
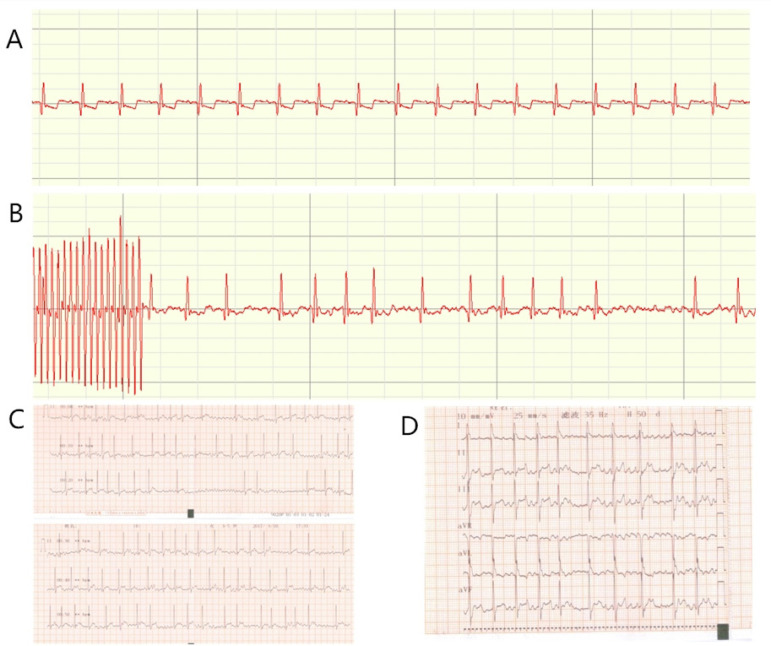
Electrocardiography (ECG). A) Baseline ECG traced by electrophysiological recorder; B) atrial fibrillation after burst stimulation traced by electrophysiological recorder; C) one-minute ECG; D) 12-lead ECG.

**Fig. 2:**
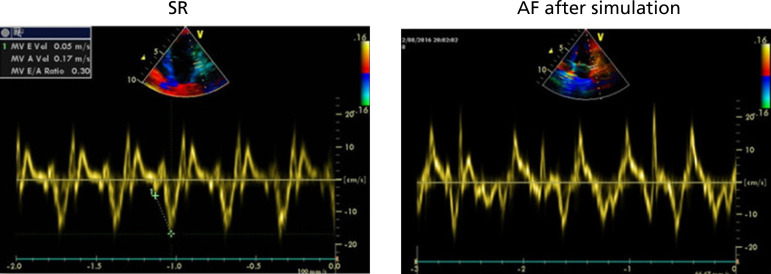
Transthoracic echocardiographies. The left image is the sinus rhythm (SR). The right image is atrial fibrillation (AF) induced by stimulation.

There were no deaths in our experiments. The ECGs showed that all canines were in sinus rhythm and did not switch to AF with the S1S2 and burst stimulations either. After eight weeks of the ATP, no canines in the ATP group were in AF. Two canines changed to AF after S1S2 stimulations, and three canines switched to AF after burst simulations. The induced-AF rate was 83.3%. The burst simulation was 10 Hz for 30 seconds. The periods of AF were 10-30 minutes. In the control group, no canines showed AF after S1S2 and burst stimulations either (Figures 1 and 2).

### Transthoracic Echocardiography Analysis

The TTE results are shown in Table 1. The baselines and eight-week TTE results were not significantly different. The left atrium (LA) diameters (from the mitral valve to the roof of the LA) in the ATP group showed differences between the baseline and the eight-week results ( *P*=0.007). In the ATP group, no canine had mitral regurgitation at the beginning. However, there were three canines with combined mitral regurgitation before sacrifice. There were one moderate regurgitation with 3.35 cm^2^ and two mild regurgitations with 1.2 cm^2^ and 1.4 cm^2^. In the control group, there were two canines with mitral regurgitation at baseline, which were 0.41 cm^2^ and 1.68 cm^2^. The mitral regurgitation areas changed to 0.34 cm^2^ and 2.25 cm^2^. The other canines did not have mitral regurgitation. The new onset of mitral regurgitation between control group and ATP group had significant difference (0 vs. 50%, personal chi-square=4.0, *P*=0.046).

**Table 1:** Comparison of transthoracic echocardiography results.

Group		ATP	Control	*P*-value
LA (long axis), mm	Baseline	23.8±6.4	23.0±1.8	0.793
	8-week	25.5±3.6	24.0±2.4	0.415
	*P*-value	0.557	0.111	
RA (four chambers), mm	Baseline	24.0±2.1	23.8±1.9	0.889
	8-week	23.0±3.0	24.8±3.1	0.317
	*P*-value	0.576	0.363	
LVED, mm	Baseline	33.5±4.5	31.7±3.6	0.452
	8-week	33.3±4.4	31.5±2.6	0.401
	*P*-value	0.895	0.822	
LVEF, %	Baseline	65.7±11.4	62.7±7.4	0.600
	8-week	55.3±4.3	61.5±8.4	0.140
	*P*-value	0.065	0.817	
LA (mitral valve to roof), mm	Baseline	23.3±1.0	25.5±3.3	0.153
	8-week	26.3±2.1	24.2±2.2	0.133
	*P*-value	0.007	0.375	
LAEDA, cm^2^	Baseline	6.5±2.1	6.7±1.1	0.915
	8-week	7.3±1.4	6.7±1.0	0.421
	*P*-value	0.088	0.758	
LAESA, cm^2^	Baseline	4.5±1.6	4.1±1.0	0.624
	8-week	4.7±0.8	4.2±0.9	0.301
	*P*-value	0.764	0.948	
ΔLA^¶^, mm		1.8±6.8	1.0±1.3	0.796

¶ means the difference between the baseline and eight-week materials of one canine.

ATP=atrial tachycardia pacing; LA=left atrium; LAEDA=left atrial end-diastolic area; LAESA=left atrial end-systolic area; LVED=left ventricular end-diastolic diameter; LVEF=left ventricular ejection fraction; RA=right atrium

### Histopathology and Immunohistochemistry

**Fig. 3:**
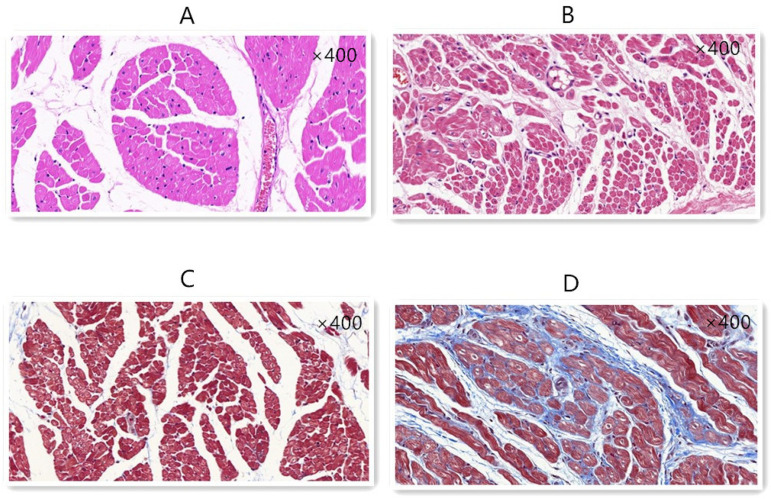
Hematoxylin-eosin (HE) and Masson’s stainings of the right atrium (×400). A) HE staining in the control group showed normal cell form and connections. B) HE staining in the atrial tachycardia pacing (ATP) group showed abnormal cell connections. There was more extracellular matrix. C) Masson’s staining in the control group. D) Compared with that of the control group, Masson’s staining in the ATP group showed significant blue fiber tissue.

HE staining and Masson’s trichrome staining were performed to determine the degree of fibrosis of the canine atrium and tissue structure. In the control group, the myocardial cells were close. However, in the ATP group, the connection of myocardial cells was loose. The collagen volume was larger in the ATP group than in the control group (Figure 3).

**Fig. 4:**
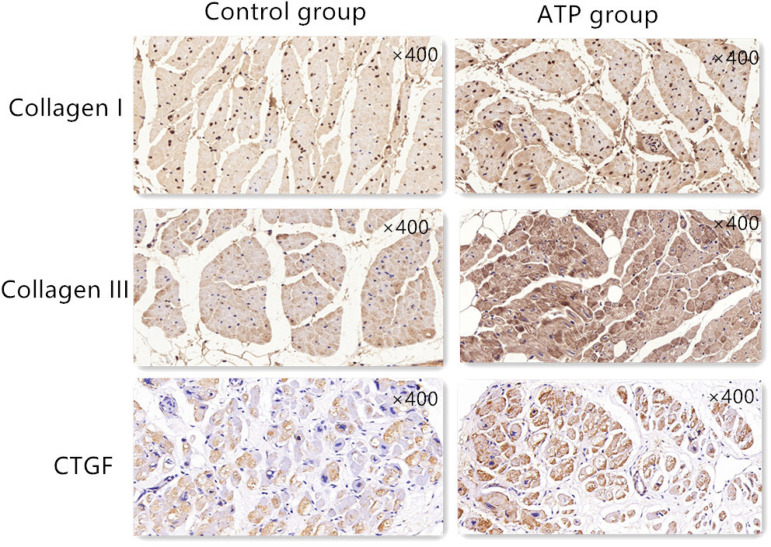
Immunohistochemistry (IHC) of the right atrium (×400). The brown colour means the target protein. The IHC showed that the collagen I, collagen III, and connective tissue growth factor (CTGF) levels in the atrial tachycardia pacing (ATP) group were significantly more evident than in the control group.

Compared with the control group, the expression of CTGF, collagen I, and collagen III detected by immunohistochemistry was significantly increased in the ATP group (Figure 4).

### Quantitative Real-time Polymerase Chain Reaction

**Fig. 5:**
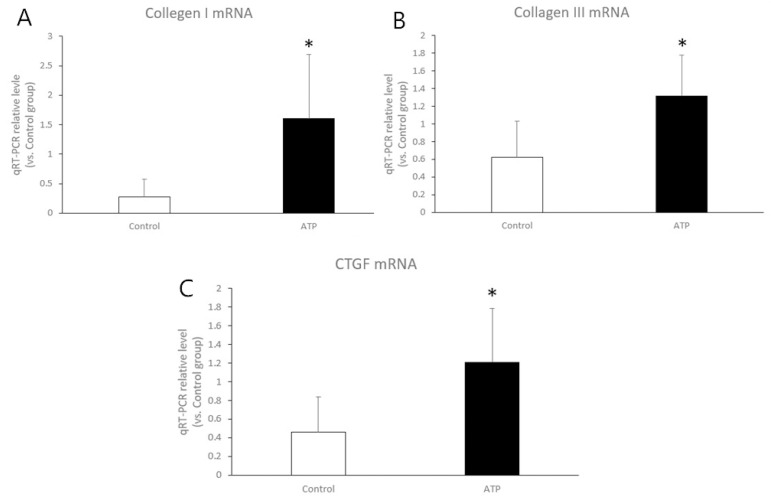
Quantitative real-time polymerase chain reaction (qRT-PCR) analysis of the messenger ribonucleic acid (mRNA) expression of collagen I (A), collagen III (B), and connective tissue growth factor (CTGF) (C) in right atrium tissue after eight weeks of atrial tachycardia pacing (ATP). Compared with those of the control group, the collagen I, collagen III, and CTGF mRNA expression levels in the ATP group were significantly increased. *Means P<0.05.

The expressions of CTGF mRNA, collagen I mRNA, and collagen III mRNA levels increased significantly in the ATP group compared with the control group (1.21±0.58 *vs*. 0.46±0.38, 1.61±1.08 *vs*. 0.28±0.30, and 1.32±0.46 *vs*. 0.62±0.41, respectively, *P*<0.05) (Figure 5).

### Western Blot Analysis

**Fig. 6:**
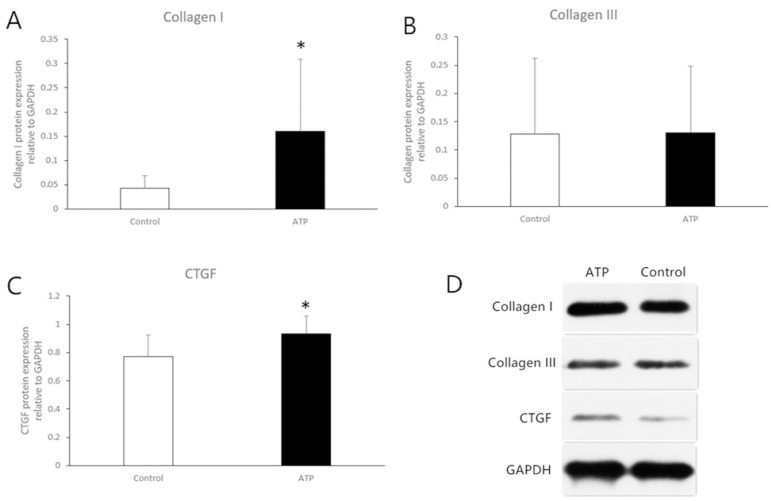
Western blot analysis of collagen I (A), collagen III (B), and connective tissue growth factor (CTGF) (C) protein levels from right atrial tissues eight weeks after the atrial tachycardia pacing (ATP). Compared with the levels in the control group, the collagen I and CTGF protein expression levels in the ATP group were significantly increased. D) Gray scale maps. *Means P<0.05. GAPDH=glyceraldehyde-3-phosphate dehydrogenase

The protein expression levels of CTGF and collagen I were significantly increased in the ATP group compared with the control group (0.9344±0.1252 *vs*. 0.7731±0.1528, *P*=0.029; 0.16±0.148 *vs*. 0.043±0.025, respectively, *P*=0.034). The protein expression of collagen III had no significant difference between the ATP and control groups (Figure 6).

## DISCUSSION

This modified beagle model of AF using a right atrium pacemaker outside the body was successful. AF was induced in five beagles (83.3%) in the ATP group. As we know, there have been many AF animal models. Different teams showed various research results using different animal models. Lee^[15]^ showed an AF-induced rate of 86% with the maintained AF time of 3.6 minutes, using an acute porcine AF model. Yu^[16]^ built a periodontitis canine model to study the relationship with AF morbidity. The author found that 41.7% and 83.3% had AF after 60 days and 90 days, respectively, which were higher than 8.3% in the control group. Lin^[17]^ built a porcine atrial tachypacing model and set the pacing electrode at the right auricle from the left jugular vein. After six weeks of ATP with 600 bpm, 91% (20/22) of porcine subjects appeared with AF, which lasted over 24 hours. Ye^[18]^ showed that the AF incidence rate was 40% with a rabbit ATP model after four weeks of left auricle tachypacing. Jan Remes built a goat CVO model to study AF and found that nine goats appeared with continued AF for over one hour and that the 12 goats were alive after four weeks^[7]^. Danshi Li^[19]^ found that the continuation of AF in a canine CHF model was longer than that of the control group. The author also found that the effective refraction period (ERP) and refractory period did not significantly change, except for the interstitial fibrosis; in chronic ATP models, different times were needed for success in different animals, at least four-eight weeks. In our modified model, we chose eight weeks for the pacing time to make sure of the effect of the ATP. There were other indexes that could be used to show the electrophysiological changes, such as the ERP and etc. Wang^[20]^ found that the ERP were shorten in the rabbit AF model induced by rapid pacing atrium and that the sarcoplasmic reticulum Ca^2+^ATPase2a (SERCA2a) overexpression was capable of suppressing it. All the final goals of these researches were useful for the treatment of AF, whatever were the reasons, the treatments, and the follow-ups. As Weymann^[21]^ found, platelet played a critical and precipitating role in the occurrence of AF. It hinted that we needed to concern the distribution width of platelets as well as factors of platelet activity. Coronary artery thromboembolism^22^ could be a nonatherosclerotic cause of acute coronary syndrome which induced AF. Our modified model also had some benefits. In our preliminary experiments, we had tried to implant the pacemakers into the subcutaneous pockets. We found that the devices suffered many complications, such as corrosion, infection, and electric leakage, which severely affected the pacemaker, aggravated adverse reactions, and even influenced the experiment results; so, we tried to put the pacemaker outside the body. At the beginning, the electrode lines were punctured out from the right lateral thoracic wall and the pacemaker was also put into the lateral pocket of clothes. However, we found that the pacemaker was easily broken by the beagle. After a few tries, we found that the connection of the back and neck was the best position to place the pacemaker. Thus, the first change of animal model was that the pacemaker was placed outside the body. Second, our modified model could decrease the experiment’s cost. The pacemakers that had been implanted were useless afterwards due to the aseptic principle, but pacemakers placed outside of the body could be used again. Every animal needed at least one pacemaker if we chose an implanted pacemaker, and the pacemaker was costly. Our pacemakers had the same effect as that of the foreign, animal pacemakers. The stimulated frequency was 5-20 Hz, the output was 0-8 mV, and the stimulated width was 2 ms. After connecting the outside pacemaker and the electrode lines, we packaged it within a zipper bag and then twined it with adhesive plaster to keep it free from water.

The relationship between atrial fibrotic changes and AF has recently been demonstrated in post-mortem and open-heart surgery histological analyses obtained from surgical specimens. Many studies are trying to determine the precise mechanism of AF and fibrosis. The ATP AF model could bring some changes in the histopathology and immunohistochemistry. HE and Masson’s stainings showed that the connection of the cells was in disorder; the extracellular matrix and fibrosis increased significantly. CTGF is one kind of multifunctional cytokine, which directly increases the synthesis, stimulates the formation of connective tissue, and inhibits the degradation of the extracellular matrix. CTGF is widely acknowledged as a very effective promoting factor for fibrogenesis and the key player in wound healing and organ fibrosis, such as the liver, kidney, and heart. The present results showed that the overexpression of CTGF, collagen I, and collagen III were definitely in the immunohistochemistry results. In addition, compared with those of the control group, the CTGF, collagen I, and collagen III mRNA levels were significantly increased in the ATP group. The Western blot results showed that compared with those of the control group, the translations of CTGF and collagen I were significantly increased in the ATP group. These results meant that the ATP triggered the AF and not only caused the electric remodelling but also the structural remodelling. This result might give us the insight that the ATP AF model can be used for research about the structural remodelling of AF, although ATP is a kind of electrical dysfunction.

## CONCLUSION

The present study demonstrated that a modified beagle model of inducible AF using a right atrium pacemaker outside the body was effective and decreased cost. This ATP AF model significantly increased CTGF and collagen I mRNA overexpression and induced atrial fibrosis.

**Table:** 

Authors' roles & responsibilities	
SY	Substantial contributions to the conception or design of the work; or the acquisition, analysis, or interpretation of data for the work; final approval of the version to be published
BM	Substantial contributions to the conception or design of the work; or the acquisition, analysis, or interpretation of data for the work; final approval of the version to be published
HL	Substantial contributions to the conception or design of the work; or the acquisition, analysis, or interpretation of data for the work; final approval of the version to be published
WL	Substantial contributions to the conception or design of the work; or the acquisition, analysis, or interpretation of data for the work; final approval of the version to be published
CW	Substantial contributions to the conception or design of the work; or the acquisition, analysis, or interpretation of data for the work; final approval of the version to be published
MY	Substantial contributions to the conception or design of the work; or the acquisition, analysis, or interpretation of data for the work; final approval of the version to be published
YY	Substantial contributions to the conception or design of the work; or the acquisition, analysis, or interpretation of data for the work; final approval of the version to be published
ZW	Substantial contributions to the conception or design of the work; or the acquisition, analysis, or interpretation of data for the work; final approval of the version to be published
